# The Effect of a Pre-consultation Tablet-Based Questionnaire on Changes in Consultation Time for First-Visit Patients With Diabetes: A Single-Case Design Preliminary Study

**DOI:** 10.7759/cureus.31624

**Published:** 2022-11-17

**Authors:** Ai Nishida, Osamu Ogawa

**Affiliations:** 1 Department of Diabetes and Endocrinology, Kameda Medical Center, Kamogawa, JPN; 2 Information Management Headquarters, Kameda Medical Center, Kamogawa, JPN

**Keywords:** pre-consultation questionnaires, first-visit patient, tablet-based questionnaire, consultation time, diabetes

## Abstract

Background and objective

It has been reported that physicians spend about 50% of their time in the outpatient department doing non-face-to-face patient work, such as charting and desk activities. Work outside of patient consultations is often considered a burden. In this study, we aimed to examine whether the use of pre-consultation tablet-based questionnaires for first-visit diabetic patients had any impact on the time spent for consultation.

Methods

The sole participant was a diabetologist with more than 20 years of experience. The time spent in the clinic was compared via a single-case experimental design (ABAB) using paper- and tablet-based questionnaires for a total of 20 first-visit diabetic patients.

Results

The median pre-clinical time without patients was significantly shorter in the tablet group than in the paper group (two minutes and 45 seconds vs. five minutes and 39 seconds; p=0.003). The median clinical time with patients in the tablet group was significantly longer than that in the paper group (19 minutes and 37 seconds vs. 11 minutes and 25 seconds; p=0.026). The total clinical time was not significantly different between the two groups (p=0.25).

Conclusions

Our results suggest that tablet-based pre-consultation questionnaires may have an impact on the allocation of time for medical examinations and improve the quality of diabetes care.

## Introduction

In Japan, it has been reported that patients have to wait for more than three hours, only to be seen for three minutes [1]. Longer actual and sensory wait times are associated with lower treatment satisfaction [2-6], and treatment satisfaction also correlates with glycemic control in patients with diabetes [7]. A reduction in actual and sensory wait times is presumed to be effective for glycemic control through treatment satisfaction, and a variety of wait time countermeasures are currently being considered. One report found that announcing wait times, apologizing for delays, and creating opportunities for people to use the wait time for something else were useful in addressing issues related to wait times [8]. However, it is reported that about 53% of physicians' time is spent face-to-face with the patient in the examination room, and the rest of their time is spent charting, working at a desk, etc. [9]. This report indicates that work other than examining patients places a heavy burden on the workforce. As described above, prolonged wait times are a problem for both patients and physicians in diabetes outpatient care. One possible way to reduce wait times, documentation time of medical records, and other non-face-to-face medical care time is to conduct a pre-consultation tablet-based medical questionnaire evaluation. Such questionnaires may reduce the burden of medical record documentation, especially that involving electronic medical records (EMR). Some reports have indicated that automated medical history-taking systems may have slightly increased clinical time and improved the quality of care in general internal medicine outpatient clinics without appointments [10]. However, it is not clear whether a pre-consultation tablet-based medical questionnaire can change the consultation time for first-visit diabetic patients. This study examined whether pre-consultation tablet-based medical questionnaires completed in the waiting room by first-visit patients with diabetes would have an impact on the consultation time with and without patients.

## Materials and methods

Study design 

**Figure 1:**
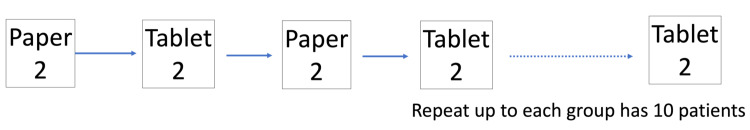
ABAB single-case design of this study Paper- and tablet-based questionnaires were used alternately in every two cases

A single-case experimental design (ABAB) without randomization or blinding was employed in this study (Figure 1).

Paper- and tablet-based questionnaires were used alternately in every two cases for diabetic patients who visited the participating physician for the first time with reservation between February 1, 2020, and May 7, 2020. A total of 20 patients were enrolled in this study. Inclusion criteria were patients who had already been diagnosed with diabetes (type 1 or type 2) and visited Kameda Medical Center for the first time.

Participant

The participant was a physician at Kameda Medical Center with over 20 years of experience as a diabetologist who agreed to participate in this study.

Medical questionnaire

The content of the medical questionnaire was selected from the initial assessment items of the comprehensive medical evaluation of the Standard of Medical Care 2020 [11].

Both paper- and tablet-based questionnaires included questions about the reason for the visit and past medical history [diabetes history including characteristics at onset (e.g., age, symptoms), previous treatment, past hospitalization, present symptoms, family history of diabetes, and lifestyle factors such as eating patterns, weight history, tobacco use, and alcohol use]. The medical questionnaire was filled out or inputted by the patients themselves in the waiting room before the consultation.

After the paper-based questionnaire was filled out, it was delivered to the examination room and reviewed by the physician before the examination, who then manually entered a summary of the information into the EMR. The results of the tablet-based questionnaire could be viewed via the Internet on a web browser in the examination room and could also be copied as text data to the EMR. This information was used by the physician as the basis for medical treatment.

Measurements, outcomes, and definitions 

Pre-clinical time without a patient, clinical time with a patient, post-clinical time without a patient, total clinical time, and total clinical time without a patient were measured. Patients’ age, gender, HbA1c at the first visit, and estimated disease duration were collected from the EMR. Measurements of consultation times were taken by the participating physician himself by using a stopwatch. The measurement times were defined as follows:

Pre-clinical Time Without Patient 

Pre-clinical time without a patient was defined as the time from when the patient’s EMR is opened until just before the patient enters the examination room.

Clinical Time With Patient 

Clinical time with a patient was defined as the time from when the patient enters the examination room till when the patient leaves. It included taking history; performing a physical examination or procedure; and assessing, planning, and discussing facts with or about a patient (family members were also considered patients). Clinical time with a patient also included computer work, documentation, and review; work done on paper or electronically; information seeking and recording details about the patient, accessing a test or image result on paper, in an EMR, in picture archiving, and in another system; asking staff about patient’s care plan, activities related to arranging medication for patients, referrals, and other nonmedication or test orders.

Post-clinical Time Without Patient 

Post-clinical time without a patient was defined as the time between when the patient leaves the examination room and when the patient's EMR is closed.

Pre-clinical time without a patient, clinical time with a patient, and post-clinical time without a patient included documentation and review; work done on paper or electronically; information seeking and recording details about the patient, accessing a test or image result on paper, in an EMR, in picture archiving, and in another system; consulting with staff, activities related to arranging medications for patients, referrals, and other nonmedication or test orders.

*Total Clinical Time* 

Total clinical time was defined as the sum of pre-clinical time without a patient, clinical time with a patient, and post-clinical time without a patient.

Total Clinical Time Without Patient 

Total clinical time without a patient was defined as the sum of pre- and post-clinical time without a patient.

Statistical analysis 

The median, minimum, and maximum time per patient for each of the two groups (the paper and tablet groups) were calculated and the differences in median time between the two groups were compared using the Mann-Whitney U test. Statistical tests were two-tailed, and a p-value <0.05 was considered statistically significant. All statistical analyses were performed using EZR (Saitama Medical Center, Jichi Medical University, Saitama, Japan), which is a graphical user interface for R (The R Foundation for Statistical Computing, Vienna, Austria). More precisely, it is a modified version of R commander designed to add statistical functions that are frequently used in biostatistics.

Ethical approval

This study was approved by the Clinical Research Review Committee of Kameda Medical Center (approval number: 18-189).

## Results

The clinical characteristics of the patients are shown in Table 1. Duration of disease and HbA1c levels did not differ significantly between the two groups (p=0.47, 0.59 respectively), while there was a significant difference in terms of age (p=0.005). The results of the Mann-Whitney U test about clinical time are shown in Table 2. Pre-clinical time without a patient was significantly shorter while clinical time with a patient was significantly longer in the tablet group. The post-clinical time without patient and the total clinical time were longer in the tablet group, but not in a statistically significant way. Total clinical time without a patient was shorter in the tablet group, also not in a statistically significant way. Total clinical time was not significantly different between the two groups.

**Table 1:** Clinical characteristics of 20 first-visit patients with diabetes in the study Data are shown as mean (range) except for gender

	Paper (n=10)		Tablet (n=10)	
		Range		Range
Age (years)	68.2	46-84	44.8	26-71
Gender				
Male (n)	4		3	
female (n)	6		7	
HemoglobinA1c (%)	7.79	5.6-11.3	8.48	5.6-15.4
Duration (years)	8.44	0-20	4.2	0-13

**Table 2:** Results of Mann-Whitney U test in paper- and tablet-based questionnaire groups Time is shown as minutes:seconds

	Paper (n=10)			Tablet (n=10)			P-value
	Median	Range	Mean rank	Median	Range	Mean rank	
Pre-clinical time without a patient	05:39	04:13-19:06	14.3	2:45	01:20-08:43	6.7	0.003
Clinical time with a patient	11:25	05:35-25:50	7.5	19:37	14:15-31:51	13.5	0.026
Post-clinical time without a patient	03:52	01:44-08:58	8.65	5:26	02:32-14:24	12.25	0.17
Total clinical time	23:52	12:14-37:35	8.9	31:37:00	15:40-34:17	12.1	0.25
Total clinical time without a patient	11:00	06:16-21:39	11.9	8:48	05:1532-20:25	9.1	0.32

## Discussion

In this study, we found that in the case of first-visit patients with diabetes mellitus, the time spent on non-face-to-face medical examinations was shorter when a tablet-based questionnaire, as opposed to a paper-based one, was used. However, the time spent on face-to-face consultations was longer when the tablet-based questionnaire was used.

Face-to-face clinic time was longer when tablet-based questionnaires were used than paper-based ones. It has been reported that a preliminary interview can reveal the patient's own issues [12] and that pre-interviews (including via the web) may deepen communication at the initial visit and enhance clinical decision-making. The use of a pre-clinical question prompt list (QPL) in breast cancer genetic counseling has been reported to be useful for assertive communication [13], and it has been noted that digital interview assistants may have a significant impact on diabetes care, including improved quality of care [14]. For these reasons, it was thought that prior information improved communication as well as face-to-face clinic time.

Tablet-based questionnaires, compared to paper-based ones, reduced non-face-to-face time before consultations. When paper-based questionnaires were used, the physician had to input the content into the EMR sequentially, whereas, with tablet-based questionnaires, the content could be copied, pasted, and edited during the consultation, which is thought to have shortened the non-face-to-face consultation time before the consultation.

Automated medical history taking has been identified as a potential way to improve the quality of care and doctor-patient interactions [15]. This study also suggests that extending face-to-face consultation time may improve both the quality of care and doctor-patient interactions. In this study, the preliminary questionnaire evaluation was conducted after the patient visited an outpatient clinic. Still, a combination of web questionnaires and online medical care is expected to improve both convenience and quality.

This study has a few limitations. There was a difference in terms of age between the two groups in this study, which may have affected the results of this study. Some EMRs have template and voice input functions. Comparing these features to tablets could yield different results. However, in terms of inputting the information prior to the clinic visit, the web-based interview is considered superior to voice or template input. This is because it is difficult for voice input and template input to be input prior to the consultation, both in online and in-person consultations. In addition, the results of this study could vary depending on the typing speed of the physician.

It is unclear whether the results can be generalized since only one physician was involved in the study and different physicians have different practice styles. Future studies quantifying typing speed in multiple physicians are necessary to validate and generalize the results of this study. Changes in quality of care and impact on glycemic control were also not evaluated. Prospective studies should be conducted in the future to address these issues.

## Conclusions

Pre-consultation tablet-based questionnaires decreased the time spent on non-face-to-face medical care prior to consultations and increased the time spent face-to-face. A change in the allocation of clinic time was observed, suggesting that this initiative could enhance the quality of care.
